# Trigeminal Neurosensory Deficit and Patient Reported Outcome Measures: The Effect on Quality of Life

**DOI:** 10.1371/journal.pone.0077391

**Published:** 2013-10-29

**Authors:** Yiu Yan Leung, Colman McGrath, Lim Kwong Cheung

**Affiliations:** 1 Oral and Maxillofacial Surgery, Faculty of Dentistry, The University of Hong Kong, Hong Kong; 2 Periodontology and Public Health, Faculty of Dentistry, The University of Hong Kong, Hong Kong; The James Cook University Hospital, United Kingdom

## Abstract

**Objectives:**

To investigate the effect of persistent neurosensory disturbance of the lingual nerve (LN) or inferior alveolar nerve (IAN) on general health and oral health- related quality of life (QoL).

**Methods:**

The study design was a case-control study. Patients with persistent neurosensory deficit of LN or IAN after lower third molar surgery (for 12 months or more) were the study group. The control group was an age and gender matched sample of patients who had dental extractions or lower third molar surgeries without trigeminal neurosensory deficit. The outcome variables were the general health and oral health-related QoL. General health-related QoL was assessed using the 36-item Short Form Health Survey (SF-36) and oral health-related QoL using the 14-item Short Form Oral Health Impact Profile (OHIP-14). Differences in SF-36 scores and OHIP-14 scores between the groups were compared.

**Results:**

Forty-eight subjects (24 cases and 24 controls) were recruited. When compared to the control group, patients with neurosensory deficits had poorer Mental-Health Component Scores (MCS) (p = 0.005), General Health (p = 0.023), Vitality (p = 0.048), Social Functioning (p = 0.003), Role-emotion (p = 0.008) and Mental Health (p = 0.022). The OHIP-14 scores were also significantly worse in this patients with neurosensory deficits compared with the control group (p = 0.002). When compared within the study group, older patient with neurosensory deficit was found to correlate with worse Physical Health Component Scores (PCS) (p = 0.02) and OHIP-14 scores (p = 0.02), while more severe visualized analog scaling rating of numbness was correlated with a worse PCS (p = 0.034).

**Conclusions:**

Patients with persistent LN or IAN deficit after lower third molar surgery have poorer health-related QoL and poorer oral health-related QoL than those without such deficits.

## Introduction

Increasingly it is recognized that patient perceptions of their oral health is important and to this end patient reported outcome measures (PROMs) are used in assessing patients’ perceptions of their health status across medicine and dentistry [1]. Neurosensory deficits are unfortunate sequelae of treatment of third molar surgery and their occurrences are not uncommon, with an estimated 0.1%–22% for lingual nerve (LN) deficit and 0.26%–8.4% for inferior alveolar nerve (IAN) deficit [2]. Neurosensory deficits may have legal consequences although it is recognized that in certain situations it may not be avoidable [3]. It is important to quantify the impact on the patients of such deficits. To date, such evidence is lacking in the literature. The consequences of neurosensory deficits after lower third molar surgery are manifold and include anaesthesia, hyperaesthesia and dysaesthesia of the area supplied by the LN or IAN, and also taste sensation. Neurosensory tests are objective assessments of the sensory change but may not fully represent the effect on one’s quality of life as they reflect symptoms rather than impact on patients’ lives.

Within medical care, the impact of health status on quality of life to assess health status and outcomes of care are now using measures such as the medical outcome survey (SF-36) for which population norm data exist for many countries [4]. In addition there are numerous oral health-related quality of life measures which provide insight into the impact of oral health status on day-to-day living or quality of life [5]. The most comprehensive measure is the Oral Health Impact Profile (OHIP) which has been widely used in many countries and across disciplines [6]. The short form measure, the 14 item OHIP has adopted well for use in the oral and maxillofacial surgery as an assessment of the need for third molar surgery as well as outcome from surgical interventions – recovery and treatment benefit [7].

The purpose of the study was to investigate the effect of persistent neurosensory disturbance of the LN or IAN on quality of life. The investigators hypothesized that patients with persistent neurosensory disturbance of LN or IAN would have a worse quality of life when compared to those who did not have the neurosensory complications. This study aimed to compare generic health-related quality of life and generic oral health-related quality of life among patients who experienced trigeminal neurosensory deficit after lower third molar surgery in a prospective case-control study, and determine the magnitude of the statistical difference. In addition the effects of demographic or neurosensory deficit-related factors of these patients on generic health-related quality of life and generic oral health-related quality of life were also investigated.

## Materials and Methods

This paper is a part of a two parts study investigating the effects of persistent trigeminal nerve deficit after lower third molar surgery on patient reported outcome measures (The other part investigates the effect on life satisfaction and depression symptoms) [8].

### Study Design and Sample

To address the research purpose, the investigators designed and implemented a case control study. The patients under reviewed in the Discipline of Oral and Maxillofacial Surgery, the Prince Philip Dental Hospital, Hong Kong with persistent or residual LN and/or IAN deficit for 12 months or more were invited to participate in this study to form the Study Group. The inclusion criteria were the patient aged 18 years or older, the neurosensory deficit of the LN and/or the IAN was a consequence of lower third molar surgery, and the neurosensory deficit confirmed by subjective and objective neurosensory tests. Subjective neurosensory assessment included a rating of their numbness by visualized analog scale (VAS) from 0 (normal) to 10 (most severely affected). Objective neurosensory assessments consisted of three tests: light touch threshold with Von Frey fibres, two-point discriminations and pain threshold. Presences of pain, hyperaesthesia or taste disturbance were also recorded. Neurosensory deficit was defined when subjective numbness VAS was greater than 0, and objective assessments are different from the unaffected side. The patients who had dental extractions or lower third molar surgeries treated in the same unit who did not present with neurosensory deficit of LN or IAN were matched with gender and age (within 2 years) with the participants of the Study Group, and were invited to participate in the study as the Control Group. The study was approved by the Institutional Review Board of the University of Hong Kong/Hospital Authority Hong Kong West Cluster (Protocol no. UW 11–451). All participants were required to provide a written informed consent.

The age, gender and education level of the participants of the study were recorded. For the participants of the Study Group, we recorded the nerve affected, the duration of the nerve injury, and if there were any pain or hyperaesthesia at the area supplied by the injured nerve. The patient’s subjective rating of numbness by visual analog scale (VAS), anchored at 0 (normal sensation) to 10 (complete anaesthesia) was recorded.

### Instruments

The study participants self-completed the Short Form Health Survey measure (SF-36) and the 14-item Oral Health Impact Profile measure (OHIP-14). The SF-36 questionnaire is a generic instrument that measures the health-related quality of life across eight domains of physical and emotional component scores. The physical component score (PCS) compose of physical functioning, role-physical, bodily pain and general health. The mental component score (MCS) compose of vitality, social functioning, role-emotional and mental health. PCS and MCS summarize the subject perceived physical health and mental health related quality of life, respectively As different SF-36 scales correlate with each of the two factors (PCS and MCS) differently, they are weighted with a physical or mental factor coefficient with a norm-based scoring *z*-score transformation in calculating the PCS and MCS [9]. The algorithm is summarized as:
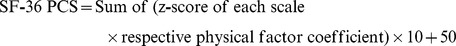


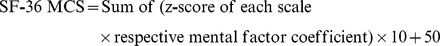



PCS, MCS and each domain range from a 0 to 100 scale, with a higher score indicates a better health status.

The OHIP-14 is across 7 domains by 14 items to assess the impact of oral health on the quality of life. The 7 domains are functional limitation, physical pain, psychological discomfort, physical disability, psychological disability, social disability and handicap. Each of the 14 items is scored 0 for “never”, 1 for “hardly ever”, 2 for “occasionally”, 3 for “fairly often”, and 4 for “very often”. The summative score can range from 0 to 56, with higher score indicating poorer oral-health related quality of life.

### Study Variables

The predictor variables were the presence of trigeminal neurosensory disturbance of a patient (Study Group versus Control Group), the demographic (age and gender) and nerve injury-related factors (numbness severity in VAS, presence or hyperaesthesia/pain, nerve involved, time lapse of nerve injury). The outcome variables were the generic health-related QoL (measured by SF-36) and oral health-related QoL (measured by OHIP-14). The primary outcome variables were the differences of the mean overall scores of SF-36 and OHIP-14 between the Study Group and the Control Group. The secondary outcomes variables were the differences of the individual domain scores of SF-36 and OHIP-14 between the Study Group and the Control Group, the differences of the mean overall scores of SF-36 and OHIP-14 of the Study Group with various demographic and nerve injury-related factors.

### Statistical Analysis

Comparison of the PCS, MCS, OHIP-14 scores and the individual domains of SF-36 and OHIP-14 between the Study/Control Group were performed using independent sample t-test. Statistical analyses were also performed to analyze the difference of PCS, MCS and OHIP-14 scores with various parameters including gender of the patients, the nerve involved, the degree of numbness, and presence of pain or hyperaesthesia on the generic and oral health-related QoL in the Study Group using paired sample t-tests. Data were analyzed with the Statistical Package for Social Sciences (SPSS version 19.0 SPSS Inc, Chicago, IL, USA). The 5% probability level was taken as the cut-off for statistical significance. The magnitude of the statistical difference was measured by calculating effect size (ES). The ES was calculated by dividing the mean scores by the pooled standard deviation, with the larger ES implying a larger difference between the two mean scores.

## Results

Forty-eight subjects were recruited. The Study Group was consisted of 24 patients (9 males) with persistent neurosensory deficit of LN (10 patients) and IAN (14 patients). The age and gender-matched Control Group was consisted of 24 patients without any neurosensory deficit. Table 1 and Table 2 show the profile of the patients of the two groups.

**Table 1:** Demographic characteristics of the study participants.

	Study Group (n = 24)		Control Group (n = 24)		
	Number	%	Number	%	p value
**Gender**					1.0
Male	9	37.5	9	37.5	
Female	15	62.5	15	62.5	
**Education**					0.731
Secondary	5	20.8	6	25	
Tertiary	19	79.2	18	75	
**Affected nerve**					
IAN	14	58.3			
LN	10	41.7			
**Mean Age (S.D)**	39.6 years	(10.8 years)	39.4 years	(10.7 years)	0.947
**Mean time of nerve injury (S.D)**	55.8 months	(57.4 months)			

**Table 2:** Neurosensory deficit characteristics of the Study Group participants (n = 24).

	Inferior Alveolar Nerve (n)	Lingual Nerve (n)	p value
**Presence of hyperaesthesia**			0.770
Yes	5	3	
No	9	7	
**Presence of Pain**			0.348
Yes	1	2	
No	13	8	
**Taste Disturbance**			
Yes		2	
No		8	
**Time lapse of nerve injury (S.D.)**	54.3 months (63.2 months)	58 months (51.4 months)	0.880
**Mean Numbness in VAS (S.D.)**	4.1 (2.2)	4.0 (2.7)	0.889

The PCS was insignificantly lower in the Study Group when compared to the Control Group (48.1 versus 50.9, p = 0.353). One of the four domains in the PCS (General Health) had significant lower score in the Study Group when compared to the Control Group. The MCS in the Study Group was significantly lower than the Control Group (41.0 versus 50.9, p = 0.005). The ES value of MCS was large (0.92). All four domains in the MCS had significant lower scores in the Study Group when compared to the Control Group (Table 3). The oral health-related QoL was also significant worse in the Study Group when compared to the Control Group. The OHIP-14 score was significantly lower in the Study Group than the Control Group (17.7 versus 8.1, p = 0.002). The scores of seven of the eight domains in OHIP-14 were also shown to be significantly worse in the Study Group and with moderate to large ES value (Table 3).

**Table 3:** Comparisons of QoL domains of the Study Group and Control Group.

	Study Group (n = 24)		Control Group (n = 24)			Cohen Effect Size**
	Mean	S.D.	Mean	S.D.	p value*	
**SF-36**						
Physical Health Score (PCS) [0–100]	48.1	8.5	50.9	5.9	0.353	0.38
Mental Health Score (MCS) [0–100]	41.0	12.3	50.7	8.3	0.005	0.92
Physical functioning	87.7	17.0	94.8	8.8	0.135	0.52
Role-physical	64.6	39.6	82.3	23.9	0.155	0.54
Bodily pain	65.9	24.6	73.5	23.0	0.288	0.32
General health	52.3	20.8	67.6	22.1	0.023	0.71
Vitality	50.0	24.5	63.8	16.1	0.048	0.67
Social functioning	69.8	24.1	88.5	15.2	0.003	0.93
Role-emotional	54.2	44.8	84.7	32.6	0.008	0.78
Mental health	62.5	21.2	76.0	15.2	0.022	0.73
**OHIP-14**						
OHIP-14 score [0–56]	17.7	11.7	8.1	8.2	0.002	0.95
Functional limitation [0–8]	2.5	1.8	1.2	1.6	0.003	0.76
Physical pain [0–8]	3.1	1.8	2.0	2.1	0.029	0.56
Psychological discomfort [0–8]	3.4	2.2	1.5	1.7	0.001	0.97
Physical disability [0–8]	1.8	1.8	1.3	1.4	0.493	0.31
Psychological disability [0–8]	3.1	2.1	1.5	1.7	0.005	0.84
Social disability [0–8]	1.7	2.2	0.3	1.0	0.001	0.82
Handicap [0–8]	2.1	2.3	0.5	1.2	0.004	0.87

* Mann-Whitney U tests.

** Effect size: >0.2 =  minimal change; 0.2–0.49 = small change; 0.5–0.8 =  moderate change; >0.8 =  large change.

When comparing the QoL of the various factors within the Study Group, there were significant correlations of age with PCS and OHIP-14 scores, with increased age correlated with a reduced PCS (Pearson correlation −0.47, p = 0.02) and an increased OHIP-14 score (Pearson correlation 0.471, p = 0.02). It was also noted the PCS scores were worse in the more severe subjectively reported numbness of the affected individual (Pearson correlation −0.434, p = 0.034). There were no statistical differences of QoL between different gender of the patients, LN or IAN involvement, and if there was any pain or hyperaesthesia of the affected area (Table 4).

**Table 4:** Comparisons of QoL of various sample factors of the Study Group.

	Mean PCS (S.D.)	p value	Mean MCS (S.D.)	p value	Mean OHIP-14 score (S.D.)	p value
**Gender**		0.730		0.467		0.972
Male (n = 9)	47.3 (12.0)		43.5 (11.1)		17.8 (13.8)	
Female (n = 15)	48.6 (6.0)		39.6 (13.2)		17.6 (10.8)	
**Nerve affected**		0.610		0.393		0.900
LN (n = 10)	49.2 (8.0)		38.4 (14.1)		17.3 (10.6)	
IAN (n = 14)	47.3 (9.1)		42.9 (11.1)		17.9 (12.8)	
**Presence of Hyperaesthesia**		0.029		0.489		0.265
Yes (n = 8)	42.8 (8.1)		38.5 (12.5)		21.5 (13.9)	
No (n = 16)	50.7 (7.7)		42.3 (12.5)		15.8 (10.4)	
**Presence of Pain**		0.383		0.430		0.115
Yes (n = 3)	44.0 (4.8)		35.6 (21.8)		27.7 (9.1)	
No (n = 21)	48.7 (8.9)		41.8 (11.1)		16.2 (11.5)	
**Age**		0.020		0.366		0.020
Pearson Correlation	−0.470		−0.193		0.471	
**Numbness Severity in VAS**		0.034		0.931		0.317
Pearson Correlation	−0.434		0.019		0.213	
**Time lapse of Nerve Injury**		0.902		0.061		0.270
Pearson Correlation	−0.027		0.389		−0.235	

Based on the significant differences of MCS of SF-36 and OHIP-14 scores of the Study Group and the Control Group, with two-sided statistical tests of 5% alpha error, the statistical power was calculated. For MCS, the power was 88%. For OHIP-14 score, the power was 90%.

## Discussion

To date assessment of the impact of trigeminal nerve deficit to patients’ lives have largely based on symptoms experience or some physical attributes [10], [11]. There is a need to consider the impact of such deficits in a more comprehensive manner employing valid and reliable standardized measures as only this will provide the opportunity to make comparisons across studies to inform evidence-based practice, or with other sequelae of a treatment.

To the best of our knowledge this study represent the first study employing standardized measure of generic health-related quality of life and generic oral health-related quality of life using measures that have performed well in other oral and maxillofacial surgery [12], [13], [14]. In our study the prevalence of IAN damage was more prevalent than LN damage which is consistent with reports of trigeminal neurosensory deficits following third molar surgery [2].

It has been proven the SF-36 PCS and MCS scales were applicable to the Chinese population in Hong Kong [15]. Morever, PCS and MCS of the control group concur with that of population norms in the population providing evidence of the suitability of the control group as a reference comparison group [4]. There was significant difference between the control group and neurosensory deficit group in terms of the MCS score and several of the domains (general health, vitality, social functioning, role-emotion and mental health). This provides evidence that trigeminal neurosensory deficit does impact on daily life and general well-being. The magnitude of the statistical difference could be best described as moderate to large based on effect size values [16]. In terms of oral health-related quality of life there were also significance difference in OHIP-14 summary scores between those with and without a neurosensory deficit. Significant difference between the case and control group was evident in six of the seven domains, and the magnitudes of the statistical difference for most parts were large – i.e. >0.80. This would suggest that in assessing the impact of neurosensory deficits either generic health or generic oral health-related quality of life measures are useful in capturing patients’ experiences, although the oral health specific measure performs better.

There were a significant correlation between severity of numbness (as rated on VAS) and PCS scores of SF-36, and the strength of the correlation was moderate. However no significant correlation was observed between patients rating of severity of ‘numbness’ and oral-health related quality of life. Presence of hyperasthesia or pain was not associated with quality of life assessments. This in part may relate to the relatively small sample size and associated statistical power. It would be useful to confirm or refute this study in other studies with larger sample size or indeed in multicentre studies. In addition, a useful research direction would be to examine the trajectory of patients’ quality of life over time since most often neurosensory deficits recover. Nonetheless it is acknowledged that for some patients they will require surgical treatment of nerve injuries, and exploration of outcome assessment including PROMs would be useful to study in ascertain patient benefits from such management approaches.

## Conclusions

This study showed patients with persistent LN or IAN deficit after lower third molar surgery have significantly poorer general health-related QoL and poorer oral health-related QoL than those without such deficits. The magnitudes of the significant differences range from moderate to large. Older patients with neurosensory deficit had a worse physical health-related QoL and oral health-related QoL, and there was also a significant correlation of a worse physical health-related QoL and subjective ratings of numbness of the patient. These findings have implications in understanding the effect of trigeminal neurosensory deficit after lower third molar surgery from a patient’s perspective. We recommend future studies to include the component of investigating patient-reported outcome measures especially on the effect on QoL on the treatment outcome of patients with trigeminal nerve injuries.
